# HIF-2α, but not HIF-1α, mediates hypoxia-induced up-regulation of *Flt-1* gene expression in placental trophoblasts

**DOI:** 10.1038/s41598-018-35745-1

**Published:** 2018-11-26

**Authors:** Tadashi Sasagawa, Takeshi Nagamatsu, Kazuki Morita, Nobuko Mimura, Takayuki Iriyama, Tomoyuki Fujii, Masabumi Shibuya

**Affiliations:** 1 Institute of Physiology and Medicine, Jobu University, Gunma, Japan; 2 Department of Obstetrics and Gynecology, The University of Tokyo, Tokyo, Japan

## Abstract

Placental hypoxia and elevated levels of circulating soluble Fms-like tyrosine kinase-1 (sFlt-1), an anti-angiogenic factor, are closely related to the pathogenesis of preeclampsia. Although sFlt-1 secretion from the placental trophoblasts is increased under hypoxic conditions, the underlying molecular mechanism remains unclear. Previously, an authentic hypoxia response element in the *Flt-1* gene promoter was shown to be a potential binding site for hypoxia-inducible factors (HIFs). Here, we investigated the roles of HIF-1α and HIF-2α in *Flt-1* gene expression in trophoblast-derived choriocarcinoma cell lines and cytotrophoblasts exposed to hypoxic conditions. In the cell lines, increased expression of *sFlt-1* splice variants and nuclear accumulation of HIF-1α and HIF-2α were observed after hypoxic stimulation. A specific small interfering RNA or an inhibitor molecule targeting HIF-2α decreased hypoxia-induced up-regulation of *Flt-1* gene expression. Moreover, in cytotrophoblasts, increased *sFlt-1* mRNA expression and elevated sFlt-1 production were induced by hypoxic stimulation. Notably, hypoxia-induced elevation of sFlt-1 secretion from the cytotrophoblasts was inhibited by silencing the *HIF-2α*, but not *HIF-1α* mRNA. These findings suggest that hypoxia-induced activation of HIF-2α is essential for the increased production of sFlt-1 proteins in trophoblasts. Targeting the HIF-2α may be a novel strategy for the treatment of preeclampsia.

## Introduction

Preeclampsia is a pregnancy-related complication clinically characterized by the new onset of hypertension and proteinuria after 20 weeks of gestation, and occurs in approximately 5% of pregnant women worldwide^1,2^. It has also become a leading cause of maternal and fetal morbidity and mortality. One of the common features of preeclampsia is elevated maternal circulatory levels of soluble Fms-like tyrosine kinase 1 (sFlt-1), an anti-angiogenic factor^3–5^. Indeed, sFlt-1 overexpression has been shown to provoke preeclampsia-like phenotypes in pregnant animals^3,6^. Furthermore, removal of sFlt-1 from the circulation of preeclamptic pregnant women by apheresis has shown to alleviate the symptoms of preeclampsia^7–9^. Accordingly, an excess of circulating sFlt-1 may partly contribute to the development and progression of preeclampsia by antagonizing the activity of vascular endothelial growth factor (VEGF) and placental growth factor (PlGF), leading to maternal endothelial dysfunction, which causes hypertension and proteinuria. However, the molecular mechanism underlying sFlt-1 up-regulation in preeclampsia remains unknown.

sFlt-1 is a truncated form of the tyrosine kinase receptor Flt-1/VEGF receptor-1 which plays important roles in angiogenesis and vasculogenesis via VEGF and PlGF signaling^10,11^. sFlt-1 is generated by alternative splicing and premature termination of *Flt-1* pre-mRNA, maintaining the 1 to 6 immunoglobulin domains of the Flt-1 extracellular ligand-binding region^12–14^. In humans, four sFlt-1 isoforms have been reported so far^13,15–17^. Among them, sFlt-1 i13 and sFlt-1 e15a are abundantly observed in the body. Notably, the former is expressed in various types of cells such as vascular endothelial cells and macrophages, while the latter is predominantly expressed in the placenta^18^. In the serum of women with preeclampsia, both sFlt-1 i13 and sFlt-1 e15a levels have been shown to be increased^19^. In addition, their mRNA expression is significantly up-regulated in the placenta of preeclamptic pregnancies compared with that in the normotensive controls^17,18,20,21^. *In situ* hybridization analysis revealed that most of the *sFlt-1 i13* and *sFlt-1 e15a* mRNAs are localized in the trophoblasts, which are fetal cells localized between the fetal and maternal blood vessels in the placental tissues^17,18,22^. Therefore, excess amounts of sFlt-1 proteins in the maternal blood are considered to be derived from the placental trophoblasts.

Placental hypoxia is widely accepted to play a key role in placental pathologies, including preeclampsia^2^. *In vitro* studies have shown that sFlt-1 production is increased under hypoxic conditions in placental villous tissue explants and primary cytotrophoblasts^23–27^. Previously, it was shown that an authentic hypoxia response element (HRE) located in the *Flt-1* gene promoter region is a potential binding site for hypoxia-inducible factors (HIFs)^28^. The α-subunits of HIFs (HIF-1α and HIF-2α) form heterodimeric transcription factors with the β-subunit (HIF-1β), and promote the transcription of numerous genes under hypoxia^29^. In the placenta of women with preeclampsia, the protein levels of HIF-1α and HIF-2α have been reported to be up-regulated^30–32^. Moreover, the expression of both these proteins has been detected in the trophoblasts of preeclamptic placenta by immunohistochemistry^32,33^. However, the relationship between HIF-α expression and *Flt-1* transcriptional up-regulation in the trophoblasts of preeclamptic placenta is not yet fully elucidated.

In this study, we investigated the roles of HIF-1α and HIF-2α in *Flt-1* gene expression under hypoxic conditions using three different human trophoblast-derived choriocarcinoma cell lines (BeWo, JAR, and JEG-3) and human primary cytotrophoblasts. We showed that HIF-2α plays a major role in the up-regulation of sFlt-1 in these cells.

## Results

### Hypoxia-induced up-regulation of *sFlt-1* mRNA expression in trophoblast-derived choriocarcinoma cell lines

**Figure 1:**
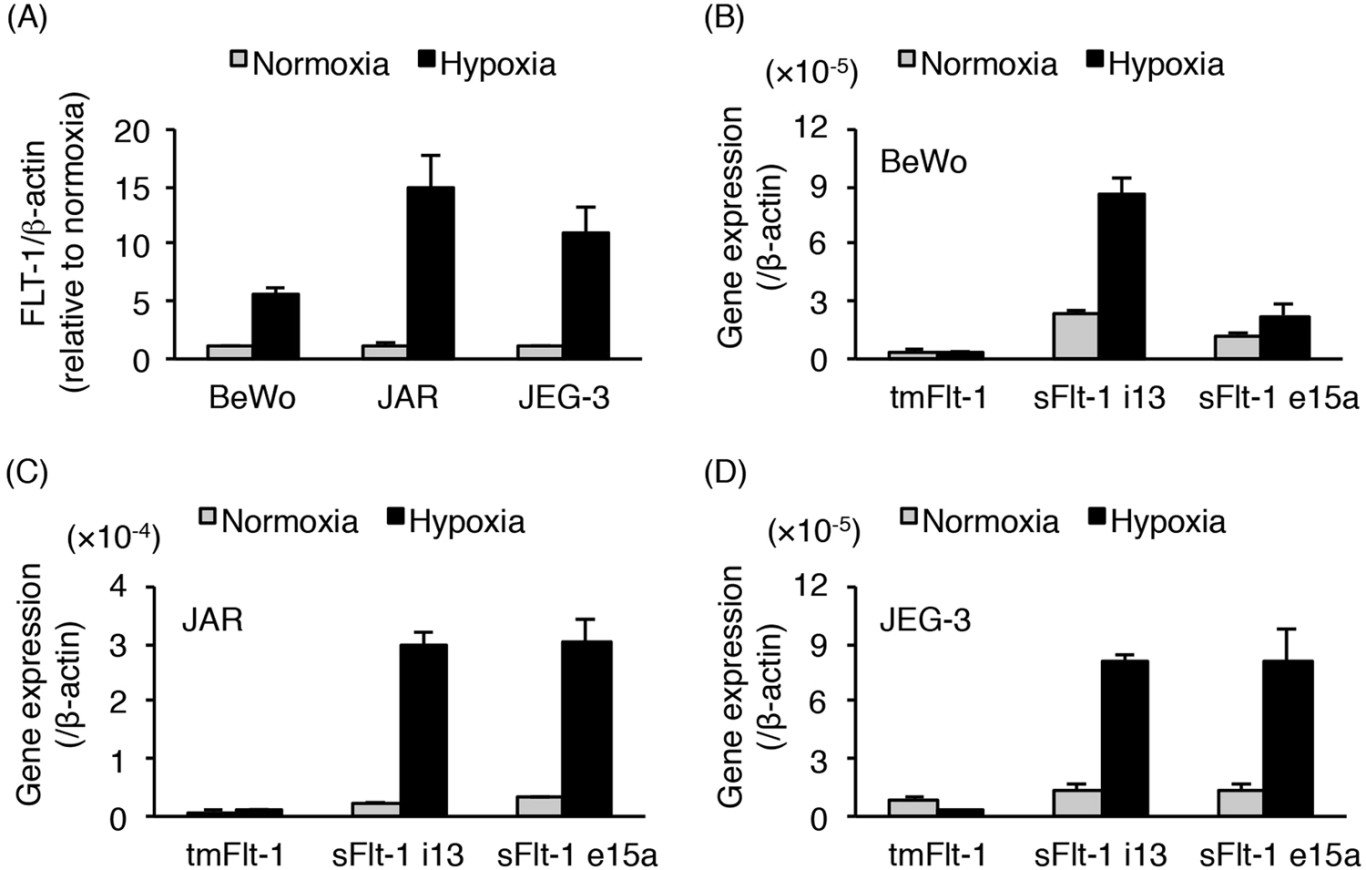
Hypoxia exposure up-regulates the expression of *sFlt-1* variants in human trophoblast-derived choriocarcinoma cell lines. BeWo, JAR, and JEG-3 cells were incubated for 24 h under normoxic or hypoxic conditions. The mRNA expression levels of all *Flt-1* transcript variants (*FLT-1*) and three splice variants of the *Flt-1* gene in these cells were determined by quantitative real-time PCR analysis using *β-actin* mRNA as a reference. (**A**) *FLT-1* mRNA expression level in the three trophoblast-derived choriocarcinoma cell lines. Results are expressed as a fold change relative to the cells under normoxic conditions. (**B**–**D**) The mRNA expression levels of three *Flt-1* splice variants (*tmFlt-1*, *sFlt-1 i13*, and *sFlt-1 e15a*) in BeWo (**B**), JAR (**C**), and JEG-3 (**D**) cells. Results are represented as a ratio relative to the expression of *β-actin* mRNA. All values are represented as the means ± SD (n = 3).

Three different human trophoblast-derived choriocarcinoma cell lines, including BeWo, JAR, and JEG-3 have been widely used as *in vitro* models for human trophoblast studies^34^. To investigate *Flt-1* expression in the three cell lines cultured under normoxic or hypoxic conditions, we first carried out quantitative real-time polymerase chain reaction (PCR) analysis using primers to cover all *Flt-1* transcript variants, including full-length transmembrane *Flt-1* (*tmFlt-1*) and *sFlt-1* mRNAs. This amplicon, *tmFlt-1* plus *sFlt-1*, was designated as *FLT-1*. The level of *FLT-1* mRNA was increased in hypoxic conditions compared to that in normoxic conditions, in all the three cell lines (Fig. 1A). We further examined the mRNA expression levels of three *Flt-1* splice variants (*tmFlt-1*, *sFlt-1 i13*, and *sFlt-1 e15a*). The mRNA levels of both *sFlt-1 i13* and *sFlt-1 e15a* were predominantly up-regulated by hypoxic stimulation in all the three cell lines, whereas the increase in *tmFlt-1* mRNA level was not clear (Fig. 1B−D). These results indicate that the hypoxia-induced increase in *FLT-1* expression level may be attributed to the increased mRNA expression of *sFlt-1* variants (*sFlt-1 i13* and *sFlt-1 e15a*).

### Activation of HIF-1α and HIF-2α in trophoblast-derived cell lines exposed to hypoxia

Since an authentic HRE is located in the *Flt-1* gene promoter region as a potential binding site for HIFs^28^, we characterized the expression and subcellular localization of HIFs in trophoblast-derived cell lines cultured under normoxia or hypoxia. Hypoxia-induced changes in protein expression and subcellular localization of HIF-1α and HIF-2α were examined by Western blot analysis and immunofluorescence staining.

**Figure 2:**
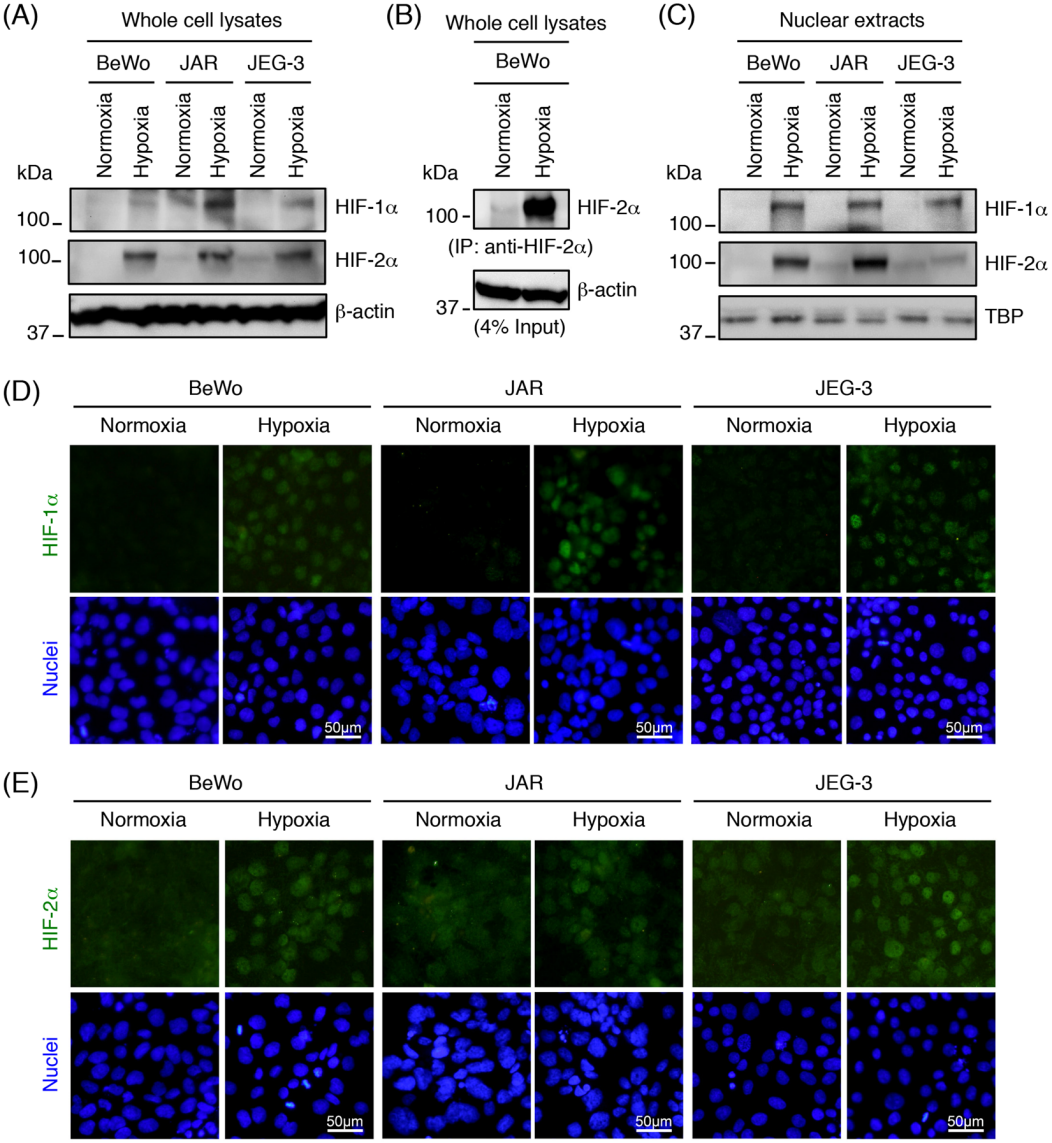
Hypoxia-induced activation of HIF-1α and HIF-2α in trophoblast-derived choriocarcinoma cell lines. Cells were incubated for 24 h under normoxic or hypoxic condition, and then whole cell lysates or nuclear extracts were prepared. (**A**) HIF-1α and HIF-2α expression in whole cell lysates derived from the three choriocarcinoma cell lines was assessed by Western blot analysis. β-actin was used as a loading control. (**B**) Immunoprecipitation (IP) of HIF-2α protein from the whole cell lysates of BeWo cells. The immunoprecipitates and whole cell lysates (input) were subjected to Western blot analysis with anti- HIF-2α and anti-β-actin antibodies, respectively. Four percent of the whole cell lysates were used as the input. (**C**) HIF-1α and HIF-2α expression in nuclear extracts derived from the three choriocarcinoma cell lines. The nuclear protein TBP served as a loading control. Uncropped images of Western blots are presented in Supplementary Fig. S5. (**D**,**E**) Cellular localization of HIF-1α (**D**) or HIF-2α (**E**) in the three choriocarcinoma cell lines was determined by immunofluorescence staining.

Western blot analysis of whole cell lysates showed that HIF-1α was only detectable when cells were exposed to hypoxia: under normoxia HIF-1α was undetectable in these cells (Fig. 2A). On the other hand, even at normoxia HIF-2α was detected at lower level in JAR and JEG-3 cells by Western blot analysis of whole cell lysates (Fig. 2A), and BeWo cells by immunoprecipitation/Western blotting (Fig. 2B). On exposure to hypoxia, both of the HIF-1α and HIF-2α protein expression were significantly increased in all the three cell lines (Fig. 2A,B). Western blot analysis showed that the HIF-1α and HIF-2α proteins were observed in the nuclear extracts after hypoxic stimulation (Fig. 2C). As demonstrated by immunofluorescence staining of cultured cells, HIF-1α was expressed in the nuclei of all three cell lines under hypoxic conditions (Fig. 2D). HIF2α displayed a cytoplasmic localization under normoxia and translocated to the nuclei after hypoxic stimulations (Fig. 2E). These results suggest that both HIF-1α and HIF-2α are activated by hypoxia exposure in all the three trophoblast-derived cell lines.

### Inhibition of HIF-2α, but not HIF-1α, decreases hypoxia-induced up-regulation of *Flt-1* gene expression in trophoblast-derived cell lines

**Figure 3:**
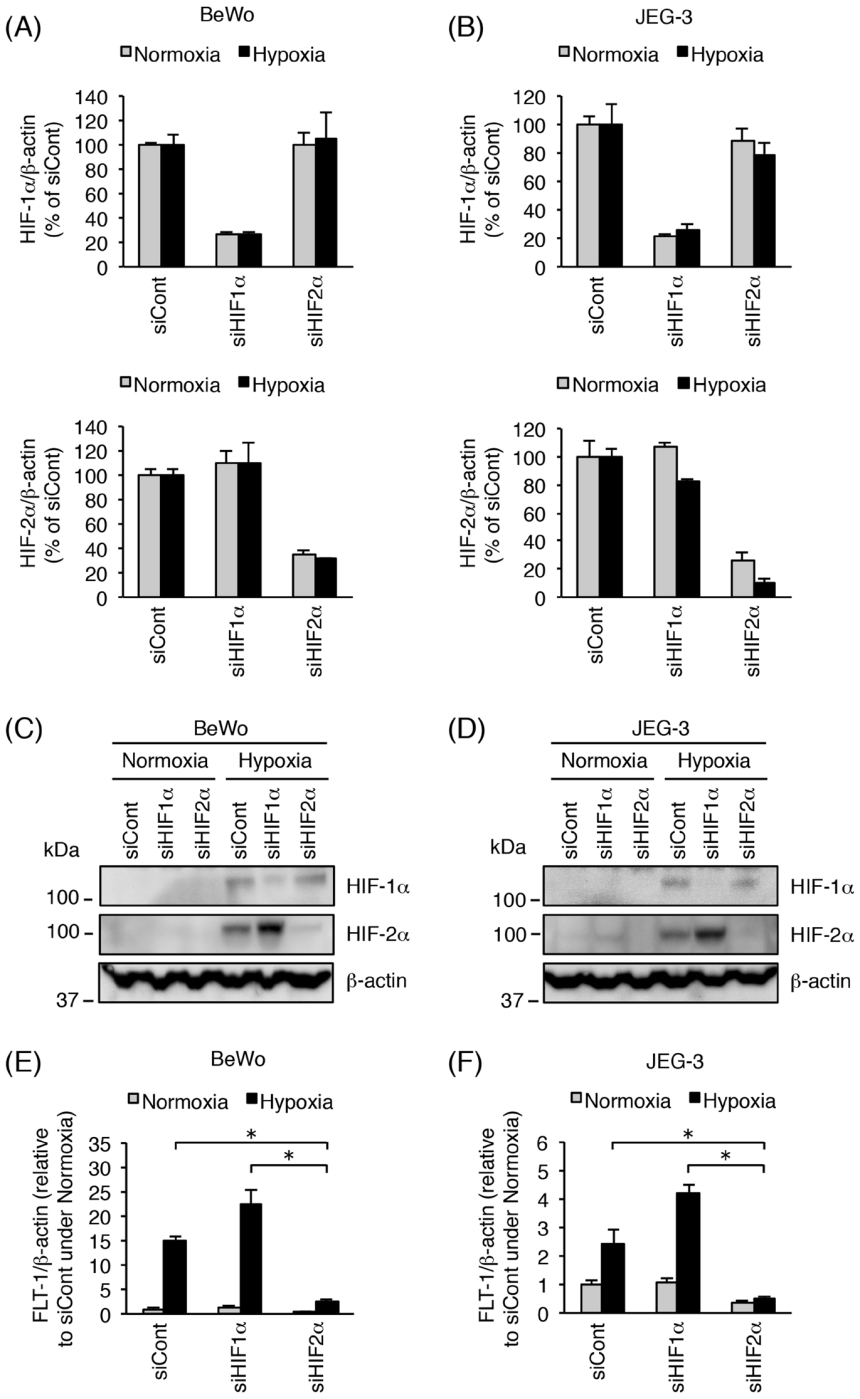
Inhibition of HIF-2α by siRNA transfection decreases hypoxia-induced up-regulation of *Flt-1* gene expression in BeWo and JEG-3 cells. BeWo and JEG-3 cells were transfected with 10 nM of control siRNA, *HIF-1α* siRNA, or *HIF-2α* siRNA. Seventy-two hours after transfection, cells were incubated for 24 h under normoxic or hypoxic conditions. (**A**,**B**) Evaluation of siRNA-mediated *HIF-1α/2α* mRNA knockdown in BeWo (**A**) and JEG-3 (**B**) cells. *HIF-1α/2α* mRNA expression levels were measured by quantitative real-time PCR analysis using *β-actin* mRNA as a reference. Results are expressed as a percentage relative to control siRNA (siCont)-transfected cells under normoxic or hypoxic conditions. (**C**,**D**) HIF-1α/2α protein expression was assessed in BeWo (**C**) and JEG-3 (**D**) cells by Western blot analysis. β-actin was used as a loading control. Uncropped images of Western blots are presented in Supplementary Fig. S5. (E, F) The mRNA expression of all *Flt-1* transcript variants (*FLT-1*) was measured in BeWo (**E**) and JEG-3 (**F**) cells by quantitative real-time PCR analysis. *FLT-1* mRNA expression level was calculated by normalizing to *β-actin* mRNA and represented as a fold change relative to control siRNA (siCont)-transfected cells under normoxic conditions. All values are represented as the means ± SD (n = 3). Asterisks indicate a significant difference (P < 0.05).

We further examined the roles of HIF-1α and HIF-2α in the up-regulation of *Flt-1* gene expression under hypoxia. BeWo and JEG-3 cells were transfected with the indicated small interfering RNAs (siRNAs) targeting *HIF-1α* or *HIF-2α* for 72 h prior to exposure to either normoxia or hypoxia for 24 h. As shown in Fig. 3A,B, specific siRNAs decreased the mRNA expression of *HIF-1α* and *HIF-2α* in both BeWo and JEG-3 cells cultured under normoxia or hypoxia. These results were confirmed at the protein level by Western blot analysis of whole cell lysates (Fig. 3C,D). Up-regulation of *FLT-1* expression was induced by hypoxic stimulation even when the cells were previously transfected with control or *HIF-1α* siRNA (Fig. 3E,F). On the other hand, transfection with *HIF-2α* siRNA significantly inhibited the hypoxia-induced up-regulation of *FLT-1* expression in both BeWo and JEG-3 cells (Fig. 3E,F).

**Figure 4:**
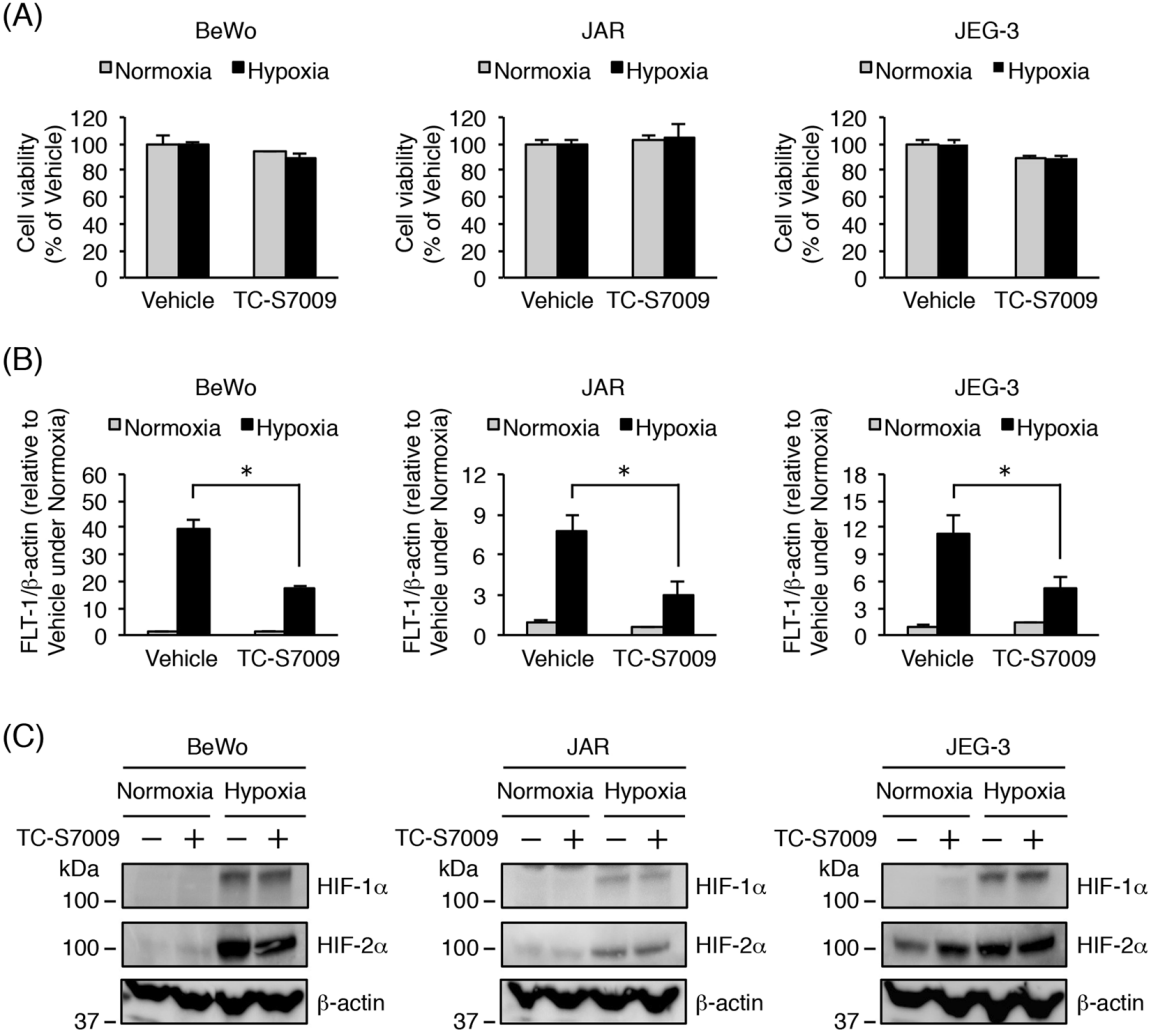
Effects of the HIF-2α inhibitor, TC-S7009, on hypoxia-induced up-regulation of *Flt-1* gene expression in trophoblast-derived choriocarcinoma cell lines. Three cell lines were cultured for 24 h under normoxic or hypoxic conditions in the presence of 0.1% DMSO (vehicle control) or 30 µM HIF-2α-specific inhibitor TC-S7009. (**A**) Cell viability was assessed to determine the potential toxicity of TC-S7009. Results are expressed as a percentage relative to vehicle-treated cells under normoxic or hypoxic conditions. (**B**) Quantitative real-time PCR analysis of the mRNA expression level of all *Flt-1* transcript variants (*FLT-1*). *FLT-1* mRNA expression level was calculated by normalizing to *β-actin* mRNA and represented as a fold change relative to vehicle-treated cells under normoxic conditions. (**C**) Western blot analysis of HIF-1α/2α protein expression levels in whole cell lysates derived from three choriocarcinoma cell lines. β-actin was used as a loading control. Uncropped images of Western blots are presented in Supplementary Fig. S5. All values are represented as the means ± SD (n = 3). Asterisks indicate a significant difference (P < 0.05).

In the case of JAR cells, the siRNA transfection conditions were not well optimized, resulting in poor efficiency of *HIF-1α* and *HIF-2α* knockdown (data not shown). Accordingly, we further investigated whether an HIF-2α-specific inhibitor, TC-S7009, was able to suppress the hypoxia-induced up-regulation of *Flt-1* gene expression in the three trophoblast-derived cell lines, including JAR. TC-S7009 is a small molecule that reduces HIF-2α-dependent gene expression by antagonizing HIF-2α heterodimerization and DNA-binding activity^35^. Treatment with TC-S7009 had no toxic effect on the three cell lines (Fig. 4A). HIF-2α inhibition with TC-S7009 significantly decreased hypoxia-induced up-regulation of *FLT-1* expression (Fig. 4B). As expected, no alteration in the protein level of HIF-1α and HIF-2α was observed in the presence of TC-S7009 (Fig. 4C). These results indicate that hypoxia-induced up-regulation of *Flt-1* gene expression is predominantly mediated by HIF-2α in all the three trophoblast-derived cell lines.

### Secretion of sFlt-1 i13 and sFlt-1 e15a is elevated under hypoxia in human primary cytotrophoblasts

**Figure 5:**
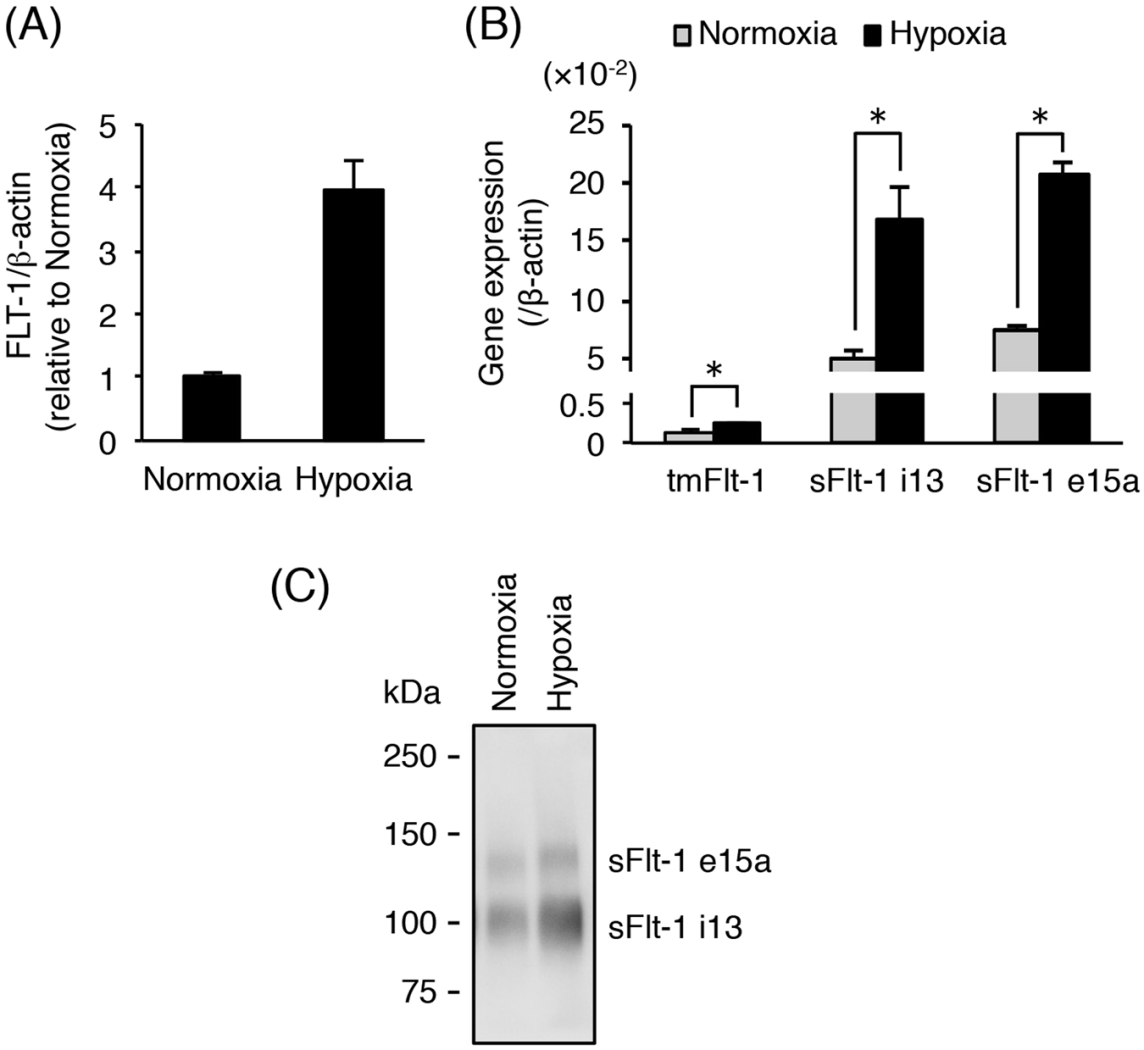
Hypoxia-induced up-regulation of sFlt-1 secretion in primary cytotrophoblasts. Thawed primary cytotrophoblasts were cultured for 16 h and then incubated for 24 h under normoxic or hypoxic conditions. Conditioned media were collected and analyzed for secretion of sFlt-1 proteins by Western blot analysis. (**A**) The mRNA expression of all *Flt-1* transcripts (*FLT-1*) in cytotrophoblasts was assessed by quantitative real-time PCR analysis using *β-actin* mRNA as a reference. Results are represented as a fold change relative to cells under normoxic conditions. (**B**) The mRNA expression levels of three *Flt-1* splice variants (*tmFlt-1*, *sFlt-1 i13*, and *sFlt-1 e15a*) in cytotrophoblasts. Results are represented as a ratio relative to the expression of *β-actin* mRNA. (**C**) Secreted sFlt-1 proteins in conditioned media were concentrated by heparin-sepharose beads and then subjected to Western blot analysis with anti-human Flt-1 N-terminal antibody. Uncropped image of Western blot is presented in Supplementary Fig. S5. All values are represented as the means ± SD (n = 3). Asterisks indicate a significant difference (P < 0.05).

The increase in sFlt-1 secretion in human primary cultured cytotrophoblasts under hypoxic condition has been previously reported^26,27^. We investigated whether the cryopreserved human primary cytotrophoblasts respond to hypoxia exposure. After thawing, the cytotrophoblasts were plated and incubated for 16 h. After changing to fresh media, the attached cells were exposed to either normoxia or hypoxia for 24 h. As shown in Fig. 5A, the *FLT-1* mRNA level was increased approximately 4-fold in the cytotrophoblasts after hypoxic stimulation. Moreover, the mRNA expression levels of *tmFlt-1*, *sFlt-1 i13*, and *sFlt-1 e15a* were significantly up-regulated on hypoxia exposure (Fig. 5B). In particular, *sFlt-1* mRNA expression was mainly increased (Fig. 5B). Hypoxia-induced elevation of sFlt-1 production was confirmed at the protein level by Western blot analysis of the conditioned media from cytotrophoblasts. To concentrate secreted sFlt-1 isoforms in the conditioned media prior to Western blot analysis, a pull-down method with Heparin Sepharose beads was used. We demonstrated that this method had a recovery efficiency of more than 85% using recombinant sFlt-1 (Supplementary Fig. S1). The protein levels of both sFlt-1 i13 and sFlt-1 e15a in the conditioned media were elevated by hypoxic stimulation (Fig. 5C). These results indicate that under our experimental condition, primary cytotrophoblasts can increase the secretion of sFlt-1 isoforms by hypoxic stimulation.

### Hypoxia-induced increase in sFlt-1 secretion is HIF-2α-dependent in human primary cytotrophoblasts

HIF-1α and HIF-2α protein levels have been shown to be increased in human primary cytotrophoblasts under hypoxic condition (2% O_2_)^33^. Using siRNA technology, we investigated whether HIF-1α or HIF-2α is involved in hypoxia-induced elevation of sFlt-1 production in cytotrophoblasts.

We first determined the optimum siRNA transfection time required to obtain a high silencing efficiency in primary cytotrophoblasts (data not shown). For example, primary cytotrophoblasts were transfected with *sFlt-1 e15a*-specific siRNA for 48 h and further incubated for 24 h under normoxic conditions. As a result, a substantial decrease in the mRNA and protein levels of sFlt-1 e15a was achieved (Supplementary Fig. S2A,B). In contrast, no significant change in *tmFlt-1* and *sFlt-1 i13* mRNA expression was detected when the cytotrophoblasts were transfected with *sFlt-1 e15a* siRNA (Supplementary Fig. S2A). In addition, no difference in sFlt-1 i13 protein production was observed (Supplementary Fig. S2B). These results indicated that our experimental condition of siRNA-mediated gene knockdown was efficient for primary cytotrophoblasts.

**Figure 6:**
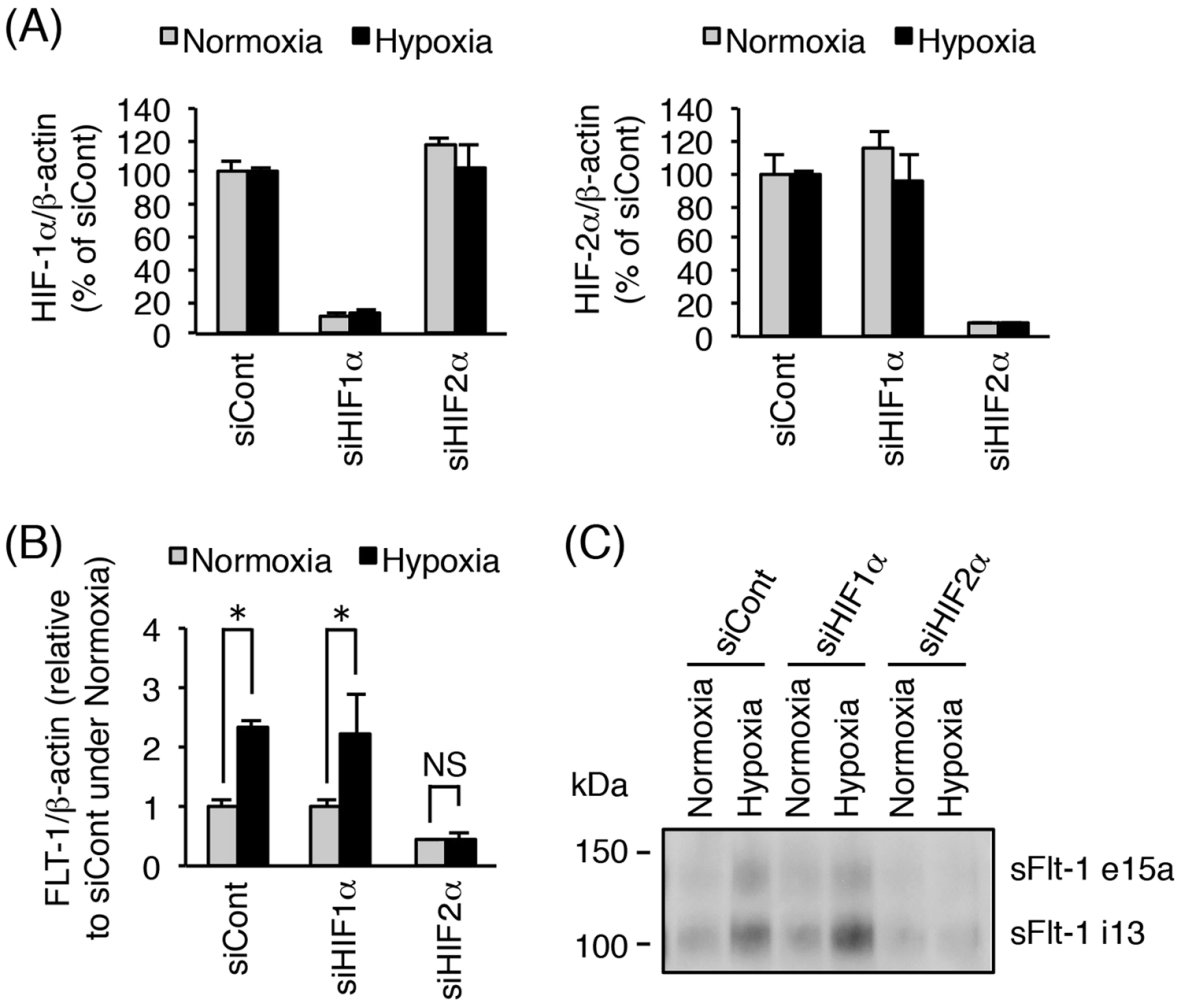
Silencing of *HIF-2α*, but not *HIF-1α*, inhibits hypoxia-induced increase in sFlt-1 secretion in primary cytotrophoblasts. Thawed primary cytotrophoblasts were cultured for 16 h and then transfected with 10 nM of control siRNA, *HIF-1α* siRNA, or *HIF-2α* siRNA. Forty-eight hours after transfection, cells were incubated for 24 h under normoxic or hypoxic conditions. Conditioned media were collected and analyzed for secretion of sFlt-1 proteins by Western blot analysis. (**A**) Evaluation of *HIF-1α/2α* mRNA knockdown by siRNA transfection. *HIF-1α/2α* mRNA expression levels were measured by quantitative real-time PCR analysis using *β-actin* mRNA as a reference. Results are expressed as a percentage relative to control siRNA (siCont)-transfected cells under normoxic or hypoxic condition. (**B**) Quantitative real-time PCR analysis of mRNA expression of all *Flt-1* transcript variants (*FLT1*). *FLT-1* mRNA expression level was calculated by normalizing to *β-actin* mRNA and represented as a fold change relative to control siRNA (siCont)-transfected cells under normoxic conditions. (**C**) Western blot analysis of sFlt-1 proteins secreted into the conditioned media. sFlt-1 proteins in the media were harvested and concentrated by heparin-sepharose beads. Uncropped image of Western blot is presented in Supplementary Fig. S5. All values are represented as the means ± SD (n = 3). Asterisks indicate a significant difference (P < 0.05). NS: No significance.

To examine the roles of HIF-1α or HIF-2α in hypoxia-induced elevation of sFlt-1 production, primary cytotrophoblasts were transfected with the indicated siRNA for 48 h prior to exposure to either normoxia or hypoxia for 24 h. No toxic effect of siRNA treatment on the primary cytotrophoblasts was observed (Supplementary Fig. S3). As shown in Fig. 6A, more than 80% knockdown of *HIF-1α* or *HIF-2α* mRNA by the respective siRNAs was achieved under both normoxic and hypoxic conditions. Next, we assessed the effect of HIF silencing on hypoxia-induced up-regulation of *Flt-1* gene expression and sFlt-1 protein production. Quantitative real-time PCR analysis revealed that *FLT-1* mRNA expression was significantly increased under hypoxia in cytotrophoblasts even when *HIF-1α* mRNA was knocked down (Fig. 6B). By contrast, when cytotrophoblasts were previously transfected with *HIF-2α* siRNA, no significant up-regulation of *FLT-1* mRNA expression was observed under hypoxic condition (Fig. 6B). Western blot analysis showed an increase in sFlt-1 i13 and sFlt-1 e15a secretion even in the presence of *HIF-1α* siRNA in cytotrophoblasts exposed to hypoxia (Fig. 6C). On the other hand, transfection with *HIF-2α* siRNA inhibited the hypoxia-induced elevation of sFlt-1 production in cytotrophoblasts (Fig. 6C). In the cytotrophoblasts derived from another donor, the respective siRNAs also achieved more than 80% knockdown of *HIF-1α* and *HIF-2α* mRNA expression (Supplementary Fig. S4A). Furthermore, *HIF-2α* siRNA, but not *HIF-1α* siRNA, specifically inhibited the hypoxia-induced up-regulation of *FLT-1* mRNA expression and elevation of sFlt-1 production (Supplementary Fig. S4B,C). These results indicate that HIF-2α, but not HIF-1α, contributes to the elevation of sFlt-1 production in primary cytotrophoblasts under hypoxia via activation of *Flt-1* gene expression at the transcriptional level.

## Discussion

In this study, using a specific siRNA or an inhibitor molecule targeting HIF-2α, we demonstrated that HIF-2α plays a major role in the hypoxia-induced up-regulation of sFlt-1 in human primary cytotrophoblasts derived from normal term placentas and human trophoblast-derived choriocarcinoma cell lines.

Among the various cells in the body, placental trophoblasts express sFlt-1 at highest amount. Other than trophoblasts, vascular endothelial cell are known to express some level of sFlt-1 in addition to tmFlt-1/VEGFR1. However, a few studies suggest that the expression level of sFlt-1 is not increased under hypoxia. Munaut *et al*. reported that the mRNA expression and protein secretion of sFlt-1 were not affected by hypoxic stimulation in human umbilical vein endothelial cells^25^. Ikeda *et al*. showed that sFlt-1 was rather down-regulated under hypoxia due to an alternative processing of mRNAs in human dermal microvascular endothelial cells^36^. Therefore, a significant increase in sFlt-1 levels in trophoblasts responding to low oxygen tension is unique among tmFlt-1/sFlt-1-expressing cells. Regarding the physiological significance of this property of trophoblasts, we consider that trophoblast-derived sFlt-1 maintains the vascular integrity of placental tissue by sequestering excess maternal VEGF produced in response to mild hypoxia.

Generally, it is widely accepted that HIF-α is up-regulated, then translocates to the nucleus, and heterodimerizes with HIF-1β under hypoxic or hypoxia-mimicking conditions to induce hypoxia-associated genes. In this process, the HIF-α/HIF-1β heterodimer binds the consensus HRE sequence 5′-(A/G)CGTG-3′ located upstream or downstream of target genes^29^. Previously, an HRE was reported to exist approximately 1,000 bp upstream of the transcription start site in mouse and human *Flt-1* genes by luciferase-based reporter assays^28,37^. However, these studies did not elucidate whether this HRE contributes to the hypoxia-induced up-regulation of *Flt-1* gene expression in trophoblasts. Further studies are necessary to assess the role of this HRE in the *Flt-1* promoter in trophoblasts exposed to hypoxia.

The selection of HIF-1α target genes, HIF-2α target genes, and HIF-1α/HIF-2α common target genes has been performed by DNA microarray analysis using RNA interference-mediated inactivation of HIF-1α or HIF-2α^38,39^. *Flt-1*, however, is not characterized as an HIF-1α and/or HIF-2α target gene. A relationship between HIF-2α and *Flt-1* expression under hypoxic condition was previously reported. In human endothelial cells, Takeda *et al*. showed that HIF-2α, but not HIF-1α, was involved in the up-regulation of *Flt-1* gene expression by chemically induced hypoxia^37^. Moreover, Eubank *et al*. reported that HIF-2α specifically regulated the hypoxia-induced sFlt-1 production in the presence of granulocyte-macrophage colony-stimulating factor in murine bone marrow-derived macrophages^40^. Consistent with previous results and our current observations, the *Flt-1* gene is considered to be one of the HIF-2α target genes. On the other hand, Okuyama *et al*. reported that HIF-1α mediates hypoxia-induced up-regulation of tmFlt-1 expression in murine bone marrow-derived mesenchymal stromal cells^41^. Nevo *et al*. showed that an *HIF-1α* antisense oligonucleotide decreased hypoxia-induced *sFlt-1* mRNA expression in human first trimester placental explants^24^. However, they did not check the efficacy of *HIF-2α* antisense oligonucleotides in this culture system. Therefore, it should be carefully studied whether the target specificity of HIF-1α and HIF-2α on the *Flt-1* gene in response to hypoxia is dependent on the cell type or developmental stage of the placenta.

Although the general mechanisms that determine the target specificity of HIF-1α and HIF-2α are still unclear, there are a few reports that attribute target specificity to the cooperation of HIF-1α and HIF-2α with other transcription factors. Knockdown of ELK-1, a member of the Ets family of transcription factors, significantly reduced hypoxic induction of the HIF-2α target genes, including *CITED2*, *WISP2*, and *IGFBP3* in MCF-7 breast cancer cells^38^. Interestingly, ELK-1 binds to an Ets-binding site adjacent to an HRE in the promoters of *CITED2* and *WISP2* genes, and also interacts with HIF-2α in MCF-7 cells exposed to hypoxia. In Hep3B hepatoma cells, upstream stimulatory factor 2 (USF2), a basic helix-loop-helix-leucine-zip transcription factor, cooperates with HIF-2α to promote the hypoxic activation of HIF-2α target genes, including *CITED2*, *EPO*, and *PAI-1*^39^. Mechanistically, USF2 activates HIF-2α target genes by interacting with HIF-2α and recruiting transcriptional co-activators, including p300 and CREB-binding protein within the HIF-2α target gene promoter. Hence, further research into the cooperative transcription factors engaged in hypoxia-induced up-regulation of *Flt-1* gene expression in trophoblasts is necessary to elucidate the mechanism of *Flt-1* gene transcriptional activation in preeclampsia.

The strategy for medical treatment of patients with preeclampsia is not yet well established. Pilot studies using apheresis to remove the sFlt-1 molecule from the circulating blood of preeclamptic pregnancies have recently been reported^7–9^. At least three strategies to decrease the circulating sFlt-1 level in patients with preeclampsia can be considered^10^: (1) a decrease in *sFlt-1* gene expression at the transcriptional level, (2) removal of the sFlt-1 protein from the blood circulation of patients with preeclampsia using a method, such as apheresis, and (3) supplementation of an appropriate amount of angiogenic factor, such as VEGF, which is trapped by the up-regulated sFlt-1 molecule. Our results strongly suggest that HIF-2α is a major factor for the stimulation of sFlt-1 expression in trophoblasts. Therefore, an efficient HIF-2α inhibitor that has no side effects on fetal growth or maternal health can be used as a promising drug for the suppression of hypertension and proteinuria, resulting in significant elongation of pregnancy period.

In conclusion, this study showed for the first time that HIF-2α, but not HIF-1α, plays a major role in hypoxia-induced up-regulation of *sFlt-1* mRNA and protein expression in human primary cytotrophoblasts. These findings may contribute not only to understanding the mechanism of excessive sFlt-1 production in patients with preeclampsia, but also to developing a novel molecular targeting therapy for the treatment of preeclampsia.

## Materials and Methods

### Trophoblast-derived choriocarcinoma cell lines and cell culture

BeWo cell line was obtained from the JCRB (Japanese Collection of Research Bioresources) Cell Bank (Tokyo, Japan). JEG-3 and JAR cell lines were purchased from the American Type Culture Collection (ATCC; Manassas, VA, USA). The three cell lines were maintained in Ham’s F-12 medium (Nacalai Tesque, Inc., Kyoto, Japan) supplemented with 10% fetal bovine serum (FBS), 100 U/mL penicillin, and 100 µg/mL streptomycin. All cells were cultured at 37 °C in a humidified atmosphere of 5% CO_2_. For hypoxia experiments, cells were seeded at a density of 4.35 × 10^4^ cells/cm^2^ in cell culture plates or dishes (Sumitomo Bakelite Co., Ltd., Tokyo, Japan). After 24 h of incubation, the medium was replaced with fresh medium, and then cells were exposed to hypoxia (2% O_2_, 5% CO_2_, and 93% N_2_) for 24 h in a multigas incubator (WAKEN 9000EX; WAKEN B TECH Co., Ltd, Kyoto, Japan).

### Isolation and culture of human villous cytotrophoblasts

All procedures involving cytotrophoblast purification from the normal term placental tissues and cytotrophoblast cultivation for several experiments were approved by the Ethical Committee of the Jobu University (No. 17-H01; http://www.jobu.ac.jp/summary/ethics_committe_minutes.html) and the institutional review board of Faculty of Medicine, the University of Tokyo (IRB number: 10580), respectively. After obtaining informed consent, normal term placentas were collected from healthy pregnant women at 37 to 40 weeks of gestation at cesarean delivery due to breech presentation and previous cesarean section. Human villous cytotrophoblasts were isolated from the villous tissues of placentas using enzymatic digestion. Percoll density gradient separation and immunodepletion with anti-HLA-ABC antibody were used to remove contaminating cells, such as placental fibroblasts, leukocytes, and extravillous trophoblast cells as previously described^33,42^. Purified cytotrophoblasts were cryopreserved in Cellbanker (ZENOAQ, Fukushima, Japan) at −80 °C until use.

Thawed primary cytotrophoblasts were suspended in a 1:1 mixture of Dulbecco’s modified Eagle’s medium (Nacalai Tesque, Inc.) and Ham’s F-12 medium containing 10% FBS and antibiotics. The cells were seeded in type I collagen-coated 12-well culture plates (Sumilon Celltight; Sumitomo Bakelite Co., Ltd., Tokyo, Japan) at approximately 1.0–1.4 × 10^6^ cells per well and incubated for 16 h. After replacing the media with fresh media, the attached cells were exposed to normoxia or hypoxia for 24 h. Conditioned media were subjected to heparin-affinity pull-down for concentrating the secreted sFlt-1 proteins, as described below.

### RNA extraction and quantitative real-time PCR

Total RNA was isolated from cells using ISOGEN reagent (Nippon Gene, Tokyo, Japan) and then reverse-transcribed to cDNA using PrimeScript RT Reagent Kit (Takara Bio, Shiga, Japan) according to the manufacturer’s instructions. Real-time PCR was performed with the Smart Cycler System (Cepheid, Sunnyvale, CA, USA) using SYBR Premix Ex Taq (Tli RNaseH Plus) (Takara Bio) or SYBR Premix Ex Taq II (Tli RNaseH Plus) (Takara Bio). The thermal cycling conditions were as follows: an initial activation cycle at 95 °C for 10 sec, followed by 40 cycles of denaturation (94 °C for 5 sec), annealing, and amplification (60 °C for 20 sec). All data were normalized to *β-actin* expression. Each value was obtained from the mean of three independent experiments. The sequences of the oligonucleotide primers are listed below: *FLT-1*, forward 5′-CCCTGTAACCATAATCATTCCGAAG-3′, reverse 5′-TCAGCCACAACCAAGGTGCTA-3′; *tmFlt-1*, forward 5′-AGAGATGGGACCGTCATCAG-3′, reverse 5′-CTGGCTCTAGCCTGCTTTTG-3′; *sFlt-1 i13*, forward 5′-ACTTGGTGCACGTTTGGATT-3′, reverse 5′-AGAGGTTGGCATCAAAATGG-3′; *sFlt-1 e15a*, forward 5′-AGTTGGAGAGCCAAGACAATC-3′, reverse 5′-CAGCATTTCACCATCTTGGTC-3′; *HIF-1α*, forward 5′-TAGAAAGCAGTTCCGCAAGC-3′, reverse 5′-ATTCATCAGTGGTGGCAGTG-3′; *HIF-2α*, forward 5′-TGTGTGAACCAATCCAGCAC-3′, reverse 5′-ACTTCATGTCCATGCTGTGG-3′; *β-actin*, forward 5′-AAATCTGGCACCACACCTTC-3′, reverse 5′-TGATCTGGGTCATCTTCTCG-3′.

### Western blot analysis

To prepare whole cell lysates, cells were lysed with RIPA buffer (50 mM Tris-HCl pH 8.0, 150 mM NaCl, 1% NP-40, 0.5% sodium deoxychorlate, and 0.1% SDS) supplemented with protease inhibitor cocktail (Nacalai Tesque, Inc.). For extracting nuclear proteins, cells were washed with cold phosphate buffered saline (PBS) and scraped in cold PBS. After centrifugation at 440 × *g* for 3 min at 4 °C, the cells were suspended in hypotonic lysis buffer (20 mM HEPES pH 7.9, 10 mM KCl, 0.2% NP-40, 10% glycerol, and protease inhibitor cocktail) and then vortexed for 10 sec. After centrifugation at 700 × *g* for 3 min at 4 °C, the pellets containing the nuclei were resuspended in nuclear extraction buffer (20 mM HEPES pH 7.9, 10 mM KCl, 350 mM NaCl, 20% glycerol, and protease inhibitor cocktail) and incubated on ice for 30 min. Supernatants (nuclear extracts) were collected by centrifugation at 17,500 × *g* for 20 min at 4 °C. The protein concentration of the whole cell lysates and nuclear extracts was determined using a protein assay kit (Bio-Rad, Hercules, CA, USA). Absorbance was measured with a DigiScan 340 T microplate reader (Asys Hitech GMBH, Eugendorf, Austria).

Whole cell lysates (70 µg protein) and nuclear extracts (30 µg protein) were separated on a SuperSep Ace gel (Wako Pure Chemicals, Osaka, Japan) and subsequently transferred onto a polyvinylidene difluoride membrane using the iBlot 2 transferring system (Invitrogen, Carlsbad, CA, USA). The following primary antibodies were used: anti-HIF-1α (1:500; Cell Signaling Technology, Beverly, MA, USA), anti-HIF-2α (1:1000; Novus Biologicals, Littleton, CO, USA), anti-β-actin (1:1000; Cell Signaling Technology), anti-TATA binding protein (TBP) (clone 58C9; 1:200; Santa Cruz Biotechnology, Santa Cruz, CA, USA), and anti-human Flt-1 N-terminal region (1:1000; provided by Dr. K. Ohsumi, Mitsubishi-Yuka BCL, Tokyo, Japan)^43^. The bands were visualized with an ECL Western Blotting Detection System (GE Healthcare, Uppsala, Sweden) on a chemiluminescence imaging system (KETA C Plus; Wealtec Corp., Sparks, NV, USA).

### Immunoprecipitation

Whole cell lysates (500 µg protein) were subjected to immunoprecipitation with Dynabeads Protein G (Invitrogen) bound to anti-HIF-2α antibody (Novus Biologicals). The precipitated proteins were subjected to Western blot analysis as described above.

### Heparin-affinity pull-down for concentrating sFlt-1 proteins

Secreted sFlt-1 isoforms in the conditioned medium from cytotrophoblasts were concentrated using a method of pull-down with Heparin Sepharose 6 Fast Flow beads (GE Healthcare). Briefly, 20 µL of bead slurry was pre-equilibrated with PBS and then incubated with 400 µL of conditioned medium at 4 °C overnight. The protein/bead complexes were washed three times with 1 mL of PBS, and the bound proteins were eluted from the beads by boiling in 15 µL of 1× SDS sample buffer (62.5 mM Tris-HCl pH 6.8, 10% glycerol, 5% β-mercaptoethanol, 2.5% SDS, and 0.01% bromophenol blue). The eluted proteins were subjected to Western blot analysis as described above.

### Immunofluorescence staining

Trophoblast-derived choriocarcinoma cell lines were cultured on FBS-coated coverslips. After incubation for 24 h under normoxic or hypoxic conditions, these cells were washed with PBS and fixed with 4% paraformaldehyde in PBS for 30 min. After being permeabilized with 0.2% Triton X-100 in PBS, the specimens were blocked with 1% bovine serum albumin (Wako Pure Chemicals) in PBS for 1 h. For immunostaining of HIF-1α and HIF-2α, the specimens were treated with anti-HIF-1α monoclonal antibody (clone H1alpha 67; 1:50; Santa Cruz Biotechnology) or anti-HIF-2α monoclonal antibody (clone ep190b; 1:400; Novus Biologicals) for 1 h, and then visualized using Alexa Fluor-conjugated secondary antibody (1:500; Invitrogen). The nuclei were counterstained with DAPI (1:500; Wako Pure Chemicals). Cell images were obtained using a fluorescence microscope (Biozero BZ-8000; Keyence, Osaka, Japan).

### siRNA transfection

Mission siRNA Universal Negative Control #1 and siRNA duplex targeting *sFlt-1 e15a* (sense 5′-CCAUUUAUUGAAAACUAUUtt-3′, antisense 5′-AAUAGUUUUCAAUAAAUGGtt-3′) were synthesized by Sigma Genosys (Hokkaido, Japan). Silencer Select Negative Control No.1 siRNA (4390843) and Silencer select validated siRNAs targeting *HIF-1α* (s6541) and *HIF-2α* (s4699) were purchased from Ambion (Austin, TX, USA). Cells were transfected with siRNAs for 48 h (cytotrophoblasts) or 72 h (trophoblast-derived cell lines) using Lipofectamine RNAiMAX transfection reagent (Invitrogen) according to the manufacturer’s instructions. Culture medium containing the siRNA and transfection reagent was changed daily during transfection. After transfection, medium was changed to fresh 10% FBS-Ham’s F12 medium, and the cells were incubated under normoxic or hypoxic conditions for an additional 24 h.

### Treatment with an HIF-2α-specific inhibitor TC-S7009

Trophoblast-derived choriocarcinoma cell lines were seeded at a density of 4.35 × 10^4^ cells/cm^2^ in cell culture plates or dishes (Sumitomo Bakelite Co.) and then cultured for 24 h. The medium was replaced with fresh medium containing 0.1% dimethyl sulfoxide (DMSO) (Sigma-Aldrich, St. Louis, MO, USA) as control vehicle or 30 μM TC-S7009 (Tocris Biosciences, Bristol, UK). After incubation for 24 h under normoxic or hypoxic conditions, the cells were subjected to cell viability assay, quantitative real-time PCR analysis, or Western blot analysis.

### Cell viability assay

Cell viability was assessed by the Cell Counting Kit-8 (CCK-8; Dojindo, Kumamoto, Japan) according to the manufacturer’s instructions. After incubation for 24 h under normoxic or hypoxic conditions, cells were incubated with CCK-8 reagent at 37 °C, followed by measurement of absorbance at 450 nm using a DigiScan 340 T microplate reader (Asys Hitech GMBH). All experiments were carried out in triplicate.

### Statistical analysis

The data are expressed as the means ± standard deviation (SD) and were analyzed by an unpaired *t*-test for parametric data. Statistical analyses were performed using Excel 2011 (Microsoft, Seattle, WA, USA) with the add-in software Statcel4 (OMS, Tokyo, Japan). A value of P < 0.05 was considered statistically significant.

### Experimental methods guideline statement

All experiments were performed in accordance with relevant guidelines and regulations.

## Electronic supplementary material

Supplemental Information
