# Supplementary material for: HIF-2α, but not HIF-1α, mediates hypoxia-induced up-regulation of Flt-1 gene expression in placental trophoblasts
Source: Sci Rep. 2018 Nov 26;8:17375. doi: 10.1038/s41598-018-35745-1 (PMC6255857; doi:10.1038/s41598-018-35745-1)
Supplement: Supplementary file 1 — Supplemental Information [file 41598_2018_35745_MOESM1_ESM.pdf]

SUPPLEMENTAL INFORMATION

HIF-2α, but not HIF-1α, mediates hypoxia-induced up-regulation of *Flt-1* gene expression in placental trophoblasts

Tadashi Sasagawa[1], Takeshi Nagamatsu[2], Kazuki Morita[2], Nobuko Mimura[2], Takayuki Iriyama[2], Tomoyuki Fujii[2], and Masabumi Shibuya[1,*]

[1]Institute of Physiology and Medicine, Jobu University, Gunma, Japan.

[2]Department of Obstetrics and Gynecology, The University of Tokyo, Tokyo, Japan.

SUPPLEMENTARY FIGURES

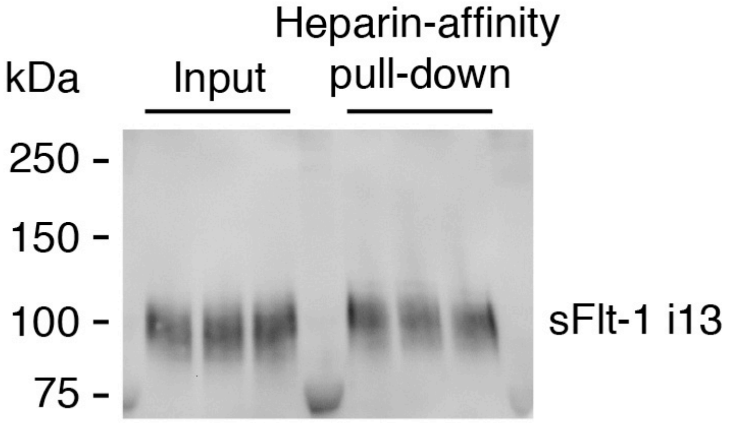

Figure S1. Recovery efficiency of recombinant sFlt-1 protein by heparin-affinity pull-down. The stable sFlt-1 i13-expressing HEK293 cells were cultured until reaching confluence. The cells were washed with serum-free medium twice and then incubated in the same medium for 2 days. The resulting conditioned medium was subjected to buffer exchange into PBS using an Amicon Ultra-0.5 centrifugal filter unit prior to heparin-affinity pull-down. In order to determine the recovery efficiency of sFlt-1 protein, 4μL (400ng protein) of conditioned medium was added to 400μL of serum-containing medium for cytotrophoblast cultivation. The samples were incubated with Heparin Sepharose beads for 3 h at 4°C. The bound proteins were eluted and then subjected to Western blot analysis.

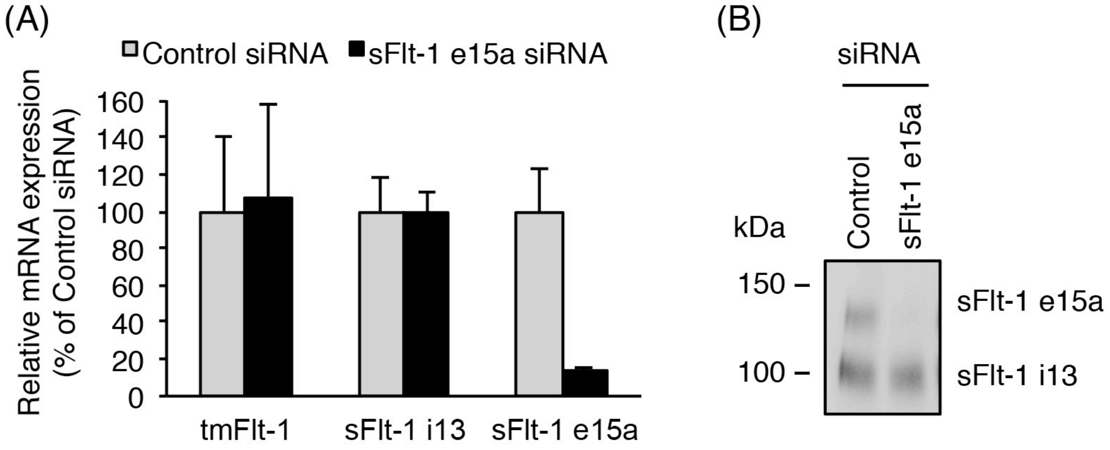

Figure S2. Effects of *sFlt-1 e15a*-specific siRNA on the mRNA expression of three *Flt-1* splice variants and secretion of sFlt-1 proteins in primary cytotrophoblasts. Primary cytotrophoblasts were transfected for 48 h with 30 nM of control siRNA or *sFlt-1 e15a* siRNA, and then incubated for 24 h under normoxic conditions. Conditioned media were collected and analyzed for secretion of sFlt-1 proteins by Western blot analysis. (A) The mRNA expression of three *Flt-1* splice variants, including *tmFlt-1*, *sFlt-1 i13*, and *sFlt-1 e15a* was determined by quantitative real-time PCR analysis using *β-actin* mRNA as a reference. Results are represented as a percentage relative to control siRNA-treated cells. (B) Western blot analysis of sFlt-1 proteins secreted into the conditioned media. sFlt-1 proteins in the media were harvested and concentrated by heparin-sepharose beads. Uncropped image of Western blot is presented in Supplementary Fig. S5. All values are represented as the means ± SD (n = 3).

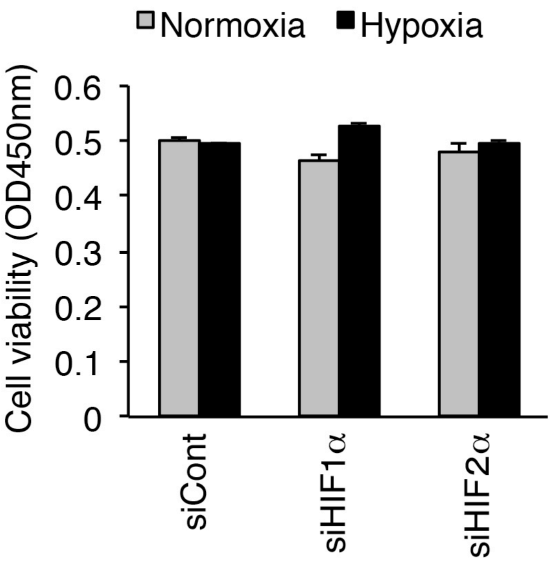

Figure S3. Effect of siRNA transfection on the viability of primary cytotrophoblasts. Primary cytotrophoblasts were transfected with 10 nM of control siRNA, *HIF-1α* siRNA, or *HIF-2α* siRNA. Forty-eight hours after transfection, cells were incubated for 24 h under normoxic or hypoxic conditions and then subjected to cell viability assay. All values are represented as the means ± SD (n = 3).

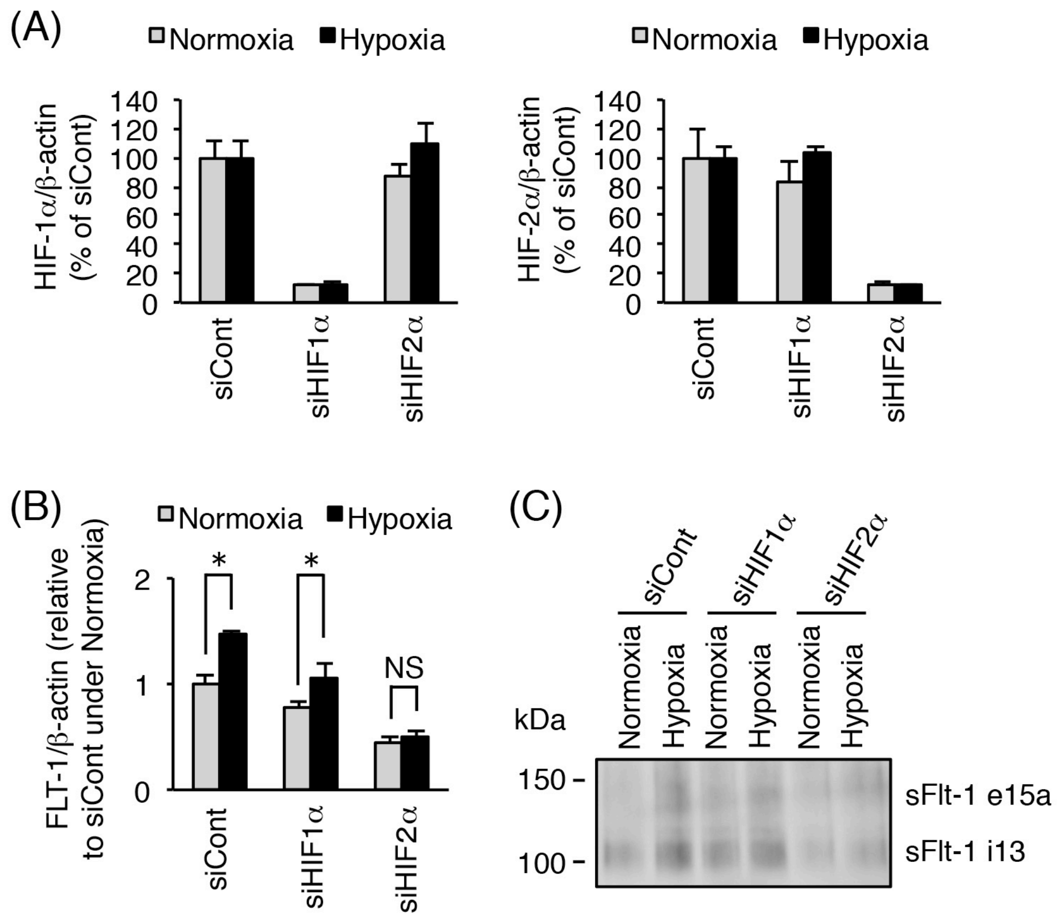

Figure S4. Silencing of *HIF-2α*, but not *HIF-1α*, inhibits hypoxia-induced increase in sFlt-1 secretion in human primary cytotrophoblasts derived from a different donor than that shown in Figure 6. Thawed primary cytotrophoblasts derived from a different donor were cultured for 16 h and then transfected with 10 nM siRNA, as indicated in this Figure. Forty-eight hours after transfection, cells were incubated for 24 h under normoxic or hypoxic conditions. Conditioned media were collected and analyzed for secretion of sFlt-1 proteins by Western blot analysis. (A, B) Quantitative real-time PCR analysis of the mRNA expression of *HIF-1α*, *HIF-2α* (A), and *FLT-1* (B). (C) Western blot analysis of sFlt-1 proteins secreted into the conditioned media. sFlt-1 proteins in the media were harvested and concentrated by heparin-sepharose beads. Uncropped image of Western blot is presented in Supplementary Fig. S5. All values are represented as the means ± SD ($n = 3$). Asterisks indicate a significant difference ($P < 0.05$). NS: No significance.

Figure 2 (A)

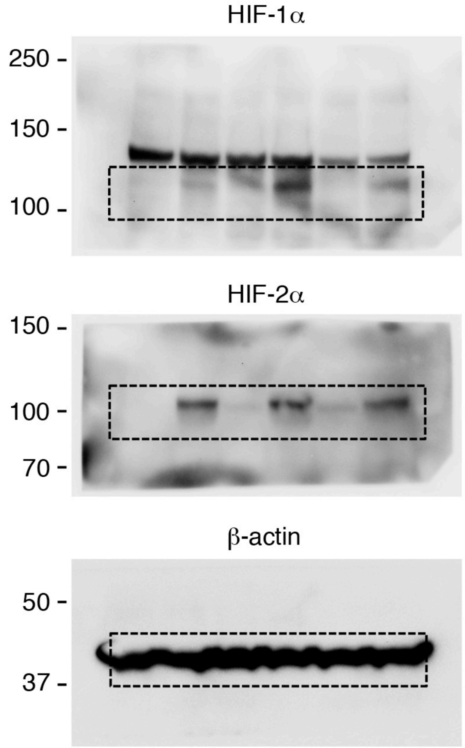

Figure 2 (B)

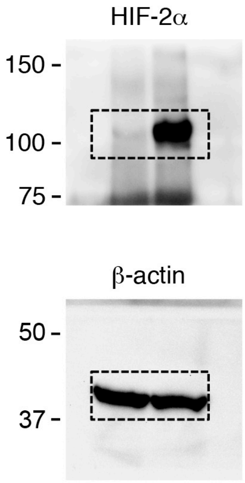

Figure 2 (C)

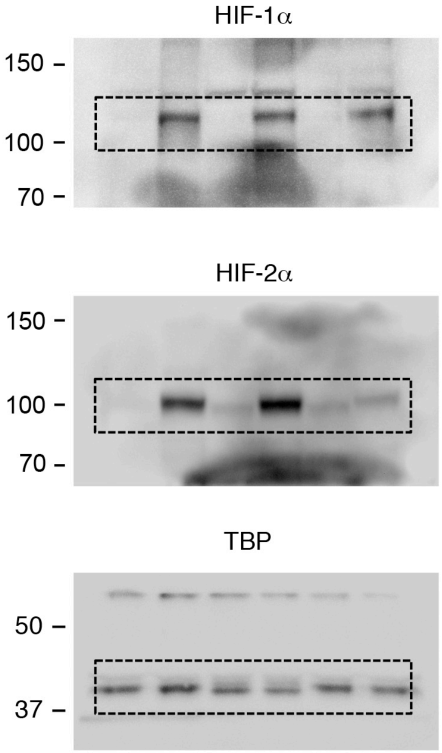

Figure 3 (C)

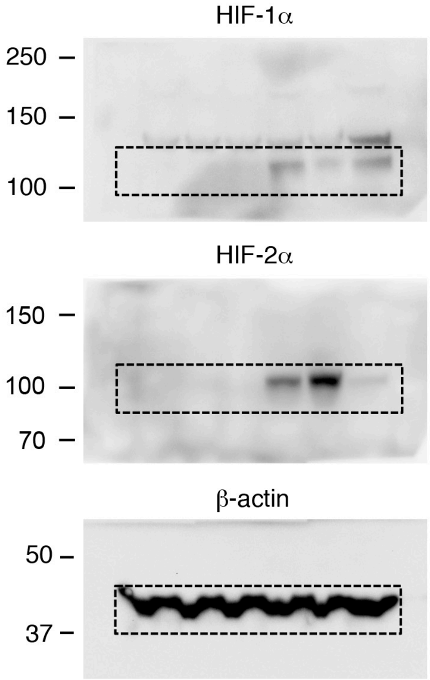

Figure 3 (D)

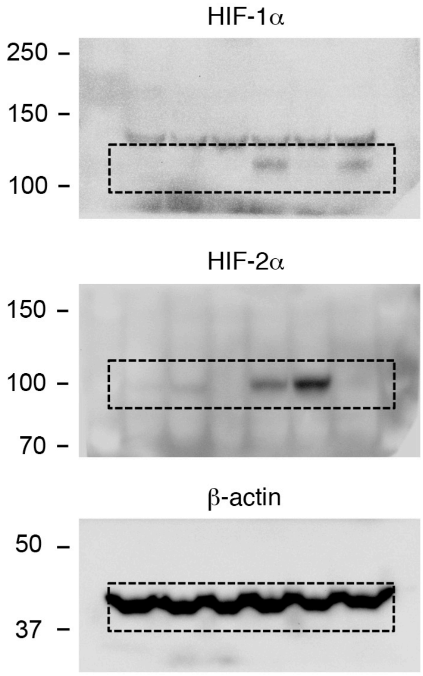

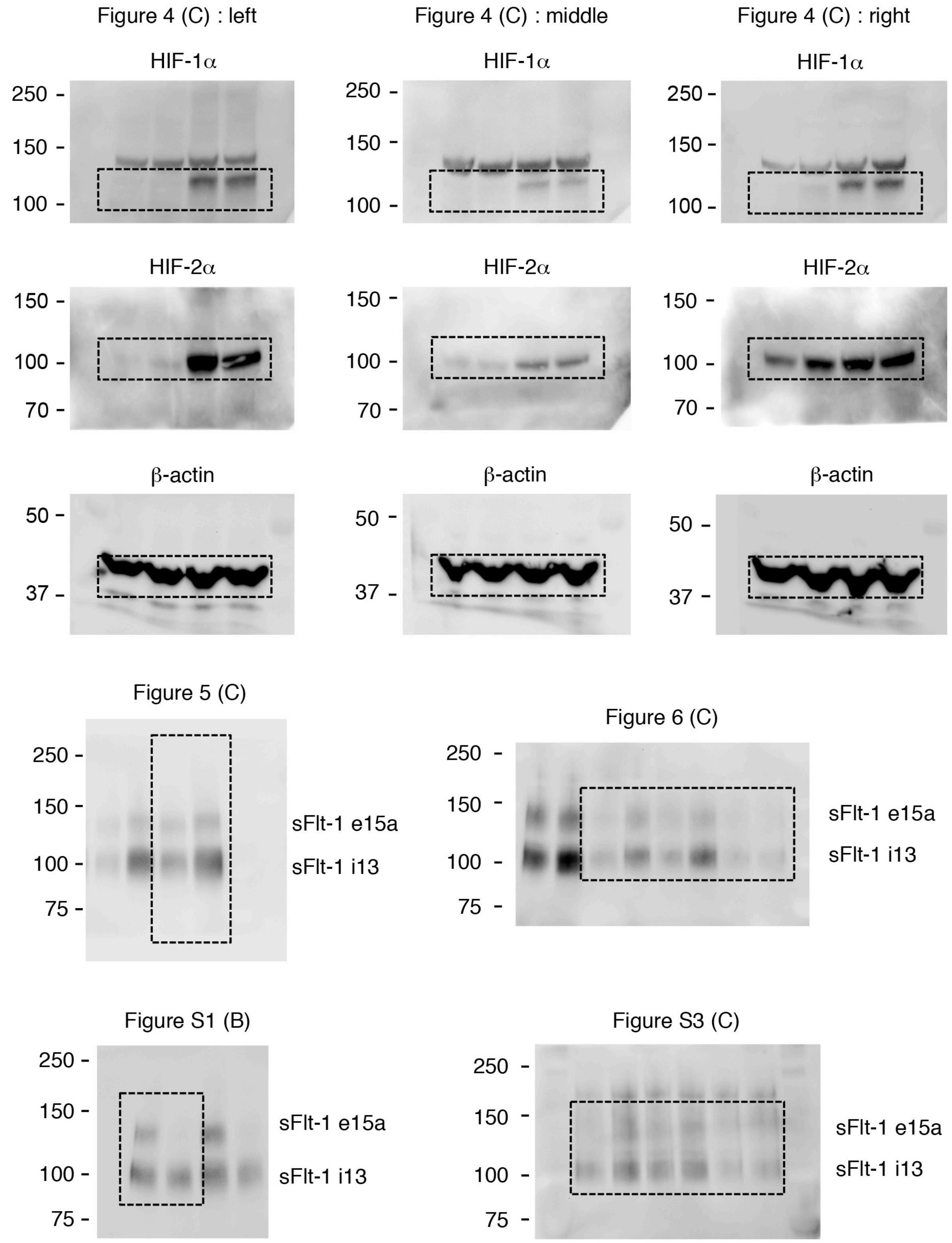

Figure S5. Uncropped images of Western blots shown in the main and supplementary figures. Boxed areas indicate the cropped regions.

SUPPLEMENTARY METHODS

Establishment of stable sFlt-1 i13-expressing HEK293 cells

To construct expression vector of sFlt-1 i13, a DNA fragment encoding the molecule were digested from pVL-6N-Flt1[1], and then cloned into bovine papilloma virus-based plasmid vector pBCMGSneo[2]. For establishment of stable sFlt1-i13-expressing human embryo kidney 293 (HEK293) cells, the vector was transfected into HEK293 cells using Lipofectamine 2000 (Invitrogen) according to the manufacturer's instructions. The transfected cells were selected in the presence of 400 µg/mL G418 (Nacalai Tesque, Inc.).

Preparation of conditioned medium from sFlt-1 i13-expressing HEK293 cells

The sFlt-1 i13-expressing HEK293 cells were cultured with Dulbecco's modified Eagle's medium (Nacalai Tesque, Inc.) containing 10% FBS, antibuitics, and 400 µg/mL G418. After the cells reached confluence, the cells were washed with serum-free medium without G418 twice and then incubated in the same medium for 2 days. The resulting conditioned medium was subjected to buffer exchange into PBS using an Amicon Ultra-0.5 centrifugal filter unit with a cutoff of 3 kDa (EMD Millipore, Hayward, CA, USA). Protein concentration of conditioned medium was determined using a protein assay kit (Bio-Rad).

SUPPLEMENTARY REFERENCES

1. Tanaka, K., Yamaguchi, S., Sawano, A. & Shibuya, M. Characterization of the extracellular domain in vascular endothelial growth factor receptor-1 (Flt-1 tyrosine kinase). Jpn J Cancer Res. **88**, 867-76 (1997)
2. Seetharam, L. *et al.* A unique signal transduction from FLT tyrosine kinase, a receptor for vascular endothelial growth factor VEGF. Oncogene **10**, 135-47 (1995)
